# Normalization of CSF pTau measurement by Aβ_40_ improves its performance as a biomarker of Alzheimer’s disease

**DOI:** 10.1186/s13195-020-00665-8

**Published:** 2020-08-15

**Authors:** Tengfei Guo, Deniz Korman, Renaud La Joie, Leslie M. Shaw, John Q. Trojanowski, William J. Jagust, Susan M. Landau, for the Alzheimer’s Disease Neuroimaging Initiative

**Affiliations:** 1 Helen Wills Neuroscience Institute, University of California, 132 Barker Hall, Berkeley, CA 94720 USA; 2 Molecular Biophysics and Integrated Bioimaging, Lawrence Berkeley National Laboratory, Berkeley, CA USA; 3 Memory & Aging Center, Department of Neurology, University of California, San Francisco, CA USA; 4 Department of Pathology and Laboratory Medicine, Perelman School of Medicine, University of Pennsylvania, Philadelphia, PA USA

**Keywords:** Tau, CSF pTau/Aβ_40_, PET, Cognition, Alzheimer’s disease

## Abstract

**Background:**

Alzheimer’s disease (AD)-related tauopathy can be measured with CSF phosphorylated tau (pTau) and tau PET. We aim to investigate the associations between these measurements and their relative ability to predict subsequent disease progression.

**Methods:**

In 219 cognitively unimpaired and 122 impaired Alzheimer’s Disease Neuroimaging Initiative participants with concurrent amyloid-β (Aβ) PET (^18^F-florbetapir or ^18^F-florbetaben), ^18^F-flortaucipir (FTP) PET, CSF measurements, structural MRI, and cognition, we examined inter-relationships between these biomarkers and their predictions of subsequent FTP and cognition changes.

**Results:**

The use of a CSF pTau/Aβ_40_ ratio eliminated positive associations we observed between CSF pTau alone and CSF Aβ_42_ in the normal Aβ range likely reflecting individual differences in CSF production rather than pathology. Use of the CSF pTau/Aβ_40_ ratio also increased expected associations with Aβ PET, FTP PET, hippocampal volume, and cognitive decline compared to pTau alone. In Aβ+ individuals, abnormal CSF pTau/Aβ_40_ only individuals (26.7%) were 4 times more prevalent (*p* <  0.001) than abnormal FTP only individuals (6.8%). Furthermore, among individuals on the AD pathway, CSF pTau/Aβ_40_ mediates the association between Aβ PET and FTP PET accumulation, but FTP PET is more closely linked to subsequent cognitive decline than CSF pTau/Aβ_40_.

**Conclusions:**

Together, these findings suggest that CSF pTau/Aβ_40_ may be a superior measure of tauopathy compared to CSF pTau alone, and CSF pTau/Aβ_40_ enables detection of tau accumulation at an earlier stage than FTP among Aβ+ individuals.

## Background

Extracellular amyloid-β (Aβ) peptides in cortical Aβ plaques and intracellular phosphorylated tau protein as neurofibrillary tangles are key hallmarks of Alzheimer’s disease (AD) that can be measured in vivo with positron emission tomography (PET) imaging and biofluid markers including plasma and cerebrospinal fluid (CSF) assays. The relationship between CSF Aβ and Aβ PET in AD has been widely reported [1–8], but relationships between CSF tau and tau PET are uncertain [9–13]. Recent studies reported that individuals with abnormal CSF phosphorylated tau (pTau) were more prevalent than individuals with abnormal tau PET only [14], and that abnormal tau PET but not CSF pTau was related to cognitive decline [15], suggesting that CSF and PET may not be interchangeable indices of tau pathology.

There are also remaining technical questions involved in measurement of CSF biomarkers. Elevated (abnormal) CSF pTau has been observed in cases with exceptionally elevated CSF Aβ_42_ in the Aβ− range [7, 16]. Positive correlations between these measurements in the Aβ− range are likely not AD-related but are instead due to individual variability in CSF production. This would suggest that abnormal CSF pTau in individuals with elevated CSF Aβ_42_ lack a pathological basis and instead reflect disease-invariant CSF increases that would be observed across all CSF markers. To address this phenomenon, use of the CSF Aβ_42_/Aβ_40_ ratio has been proposed over CSF Aβ_42_ alone [6, 7, 17–19], since Aβ_40_ is most abundant Aβ species in CSF [19, 20], and expected to increase due to higher overall Aβ production but not sensitive to AD [21–29]. We hypothesize that a similar adjustment of CSF pTau using CSF Aβ_40_ may reduce noise and improve associations with other biomarkers.

In this study, we used Alzheimer’s Disease Neuroimaging Initiative (ADNI) participants to explore the utility of a CSF pTau/Aβ_40_ ratio to reduce noise in pTau measurements and improve associations with downstream markers of AD progression. We then examined the biological plausibility of this biomarker in relation to regional ^18−^Flortaucipir (FTP) PET as well as subsequent tau PET and cognitive changes.

## Methods

### Participants

Data used in this study were obtained from the ADNI database (ida.loni.usc.edu; specific datasets used in this study are named below). The ADNI study was approved by institutional review boards of all participating centers, and written informed consent was obtained from all participants or their authorized representatives. In total, 219 cognitively unimpaired (CU) elderly adults, 91 mild cognitive impairment (MCI), and 31 AD patients with concurrent (acquisition interval within 1 year) Aβ PET (^18^F-florbetapir (FBP) or ^18^F-florbetaben (FBB)), CSF Aβ_40_, Aβ_42_ and pTau_181_, FTP tau PET, structural MRI, and cognitive test were included in this study.

### PET and MRI imaging

PET data was acquired in 5-min frames from 50 to 70 min (FBP), 90–110 min (FBB), and 75–105 min (FTP) post-injection (http://adni-info.org). PET and structural MRI scans were downloaded from the Laboratory of NeuroImaging (LONI) (ida.loni.usc.edu) and processed with Freesurfer V5.3.0. All fully pre-processed PET scans were co-registered to the structural MRI scan that was closest in time to the baseline PET. Regions of interest (ROIs) were defined on each structural MRI scan using Freesurfer (V5.3.0) and used to extract regional FBP, FBB, and FTP measurements from the co-registered PET images as described previously [30, 31].

Briefly, FBP or FBB standardized uptake value ratios (SUVRs) were calculated by dividing frontal, cingulate, parietal, and temporal regional uptake to that in the whole cerebellum to generate COMPOSITE SUVRs [30]. COMPOSITE SUVRs for FBP ≥1.11 or FBB ≥1.08 were defined as Aβ+ as described on the ADNI website. Aβ positivity was defined by Aβ PET in this study. FBP (UCBERKELEYAV45_05_12_20.csv) and FBB (UCBERKELEYFBB_05_12_20.csv) SUVRs were converted to Centiloids using the equations Centiloid = (196.9 × SUVR_FBP_) − 196.03 for FBP and Centiloid = (159.08 × SUVR_FBB_) − 151.65 for FBB (ADNI_Centiloid_Methods_Instruction_20181113.pdf in LONI website (ida.loni.usc.edu)).

For FTP (BERKELEYAV1451_05_12_20.csv), composite Temporal-metaROI (including entorhinal, parahippocampal, fusiform, amygdala, inferior temporal, and middle temporal) [32] and entorhinal cortex SUVRs were calculated using inferior cerebellar cortex intensity normalization [31]. To define FTP SUVR thresholds, we carried out ROC analyses with Temporal-metaROI and entorhinal SUVR values using the Youden index classifying 280 Aβ PET− ADNI CU participants and 183 Aβ PET+ ADNI MCI and AD patients as the endpoint (Supplemental Figs. 1–4). This resulted in a threshold of 1.25 for the Temporal-metaROI and 1.21 for entorhinal cortex. Among these 463 ADNI participants for the definition of tau PET cutoffs, 217 (47%) participants were included in the following analyses of this study. We also examined alternative thresholds for these regions defined by the mean + 2SD of 280 Aβ PET- ADNI CU participants. These resulted in more conservative thresholds of 1.34 for the Temporal-metaROI and 1.31 for entorhinal cortex. In total, 34% of 341 participants had longitudinal FTP data. FTP slope (ΔFTP, SUVR units per year) was calculated based on longitudinal FTP data for each individual using linear mixed effects (LME) model, including the following independent variables: time, APOE-ε4 status, age and gender, and a random slope and intercept. Since white matter intensity normalization has shown less variability for longitudinal tau PET changes [33–35], we calculated FTP slopes using a white matter reference region.

Hippocampal volume (HCV) (mm^3^) was calculated across hemispheres from the structural MRI scan that was closest in time to the baseline PET scan and for subsequent MRI scans using Freesurfer, and adjusted by estimated intracranial volume (ICV) using the regression approach [36]: adjusted HCV (aHCV) = HCV − 0.0017 × (ICV – 1498858), where 0.0017 and 1498858 represent the correlation coefficient between HCV and ICV, and the mean of ICV in Aβ− 323 ADNI CU participants. In total, 41% of 341 participants had longitudinal aHCV data. aHCV slope (ΔaHCV, mm^3^ units per year) was calculated based on longitudinal aHCV data for each individual using LME model, including the following independent variables: time, APOE-ε4 status, age, gender and education, and a random slope and intercept.

### CSF Aβ_40_, Aβ_42_, and pTau

CSF Aβ_40_, Aβ_42_, and pTau were analyzed by the University of Pennsylvania ADNI Biomarker core laboratory using the fully automated Roche Elecsys and cobas e 601 immunoassay analyzer system [16, 37]. CSF data (UPENNBIOMK10_07_29_19.csv) were downloaded from ADNI website. A threshold for abnormal CSF pTau was defined as ≥22 pg/mL based on an ROC analysis using the Youden index classifying 320 Aβ PET− ADNI CU participants and 429 Aβ PET+ ADNI MCI and AD patients as the endpoint (Supplemental Figs. 5–6). We also defined an alternative threshold of ≥31 for CSF pTau which was based on the mean + 2SD of CSF pTau in 320 Aβ PET− ADNI CU participants. We calculated the CSF pTau/Aβ_40_ ratio threshold as ≥0.0012 according to the same ROC approach classifying 169 Aβ PET− CU participants and 161 Aβ PET+ MCI and AD patients as the endpoint (Supplemental Figs. 7–8), and the alternative threshold was ≥0.0014 based on the mean + 2SD of the CSF pTau/Aβ_40_ ratio in 169 Aβ PET− ADNI CU participants. Among these 749 ADNI participants for the definition of CSF pTau, 212 (28%) participants were included in the following analyses of this study. Among these 329 ADNI participants for the definition of CSF pTau/Aβ_40_, 201 (61%) participants were included in the following analyses of this study.

### Cognition

The Delayed Recall portion of the Alzheimer’s Disease Assessment Scale (ADASSCORES.csv and ADAS_ADNIGO23.csv downloaded at April 28, 2020), the delayed recall score on the logical memory IIa subtest from the Wechsler Memory Scale, the digit symbol substitution test score from the Wechsler Adult Intelligence Scale–Revised (NEUROBAT.csv downloaded at April 28, 2020), and the MMSE total score (MMSE.csv downloaded at April 28, 2020) were transferred to standard z scores (using the mean values of ADNI CU participants). Preclinical Alzheimer Cognitive Composite (PACC) scores [38] were calculated by combining these 4 cognitive z scores to one composite score. In total, 59% of 341 participants had longitudinal PACC data. PACC slope (ΔPACC) was calculated for each participant based on longitudinal PACC scores using LME model, including the following independent variables: time, APOE-ε4 status, age, gender and education, and a random slope and intercept.

### Statistical analysis

Normality of distributions was tested using the Shapiro-Wilk test and visual inspection of data. Data are presented as median (interquartile range (IQR)) or number (%). Baseline characteristics were compared between Aβ− and Aβ+ groups by using a two-tailed Mann-Whitney test or Fisher’s exact test.

In order to evaluate the feasibility of using CSF pTau/Aβ_40_ as an alternative to CSF pTau, we first used generalized linear models (GLM) to examine the relationships of CSF Aβ_40_ with Aβ PET and tau PET to confirm that CSF Aβ_40_ is not related to AD biomarkers, and subsequently investigated the cross-sectional associations between CSF Aβ_42_, pTau and pTau/Aβ_40_, and controlling for APOE-ε4 status, diagnosis, sex, and age. A false discovery rate of 0.05 using the Benjamini-Hochberg approach was employed for 35 regions.

**Fig. 1:**
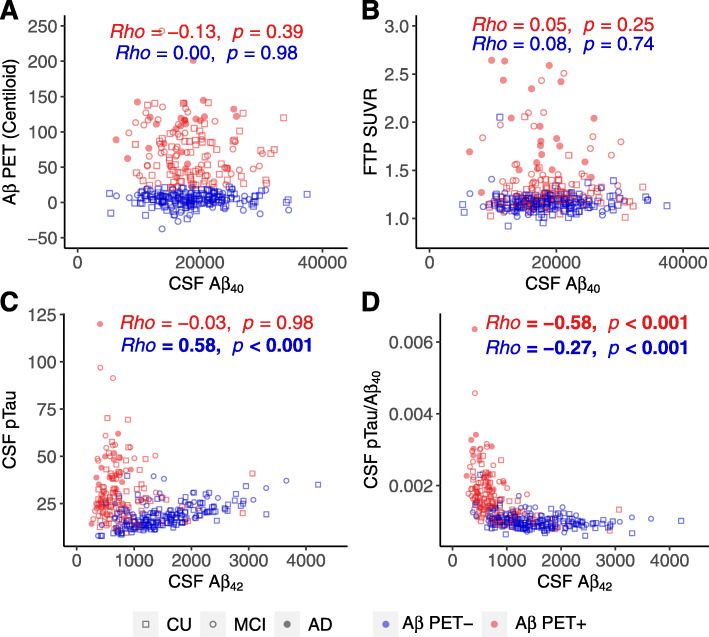
Cross-sectional associations between CSF Aβ_42_, pTau, and pTau/Aβ_40_. Associations of **a** Aβ PET (Centiloid) and **b** tau PET (Temporal-metaROI FTP SUVR) with CSF Aβ_40_. Associations between CSF Aβ_42_ and **c** CSF pTau and **d** CSF pTau/Aβ_40_. The horizontal gray dashed lines reflect the abnormal thresholds of corresponding biomarkers on *y*-axis. Abbreviations: pTau = phosphorylated tau; Aβ = amyloid-β; FTP = ^18^F-flortaucipir; CU = cognitively unimpaired; MCI = mild cognitive impairment; AD = Alzheimer’s disease

**Fig. 2:**
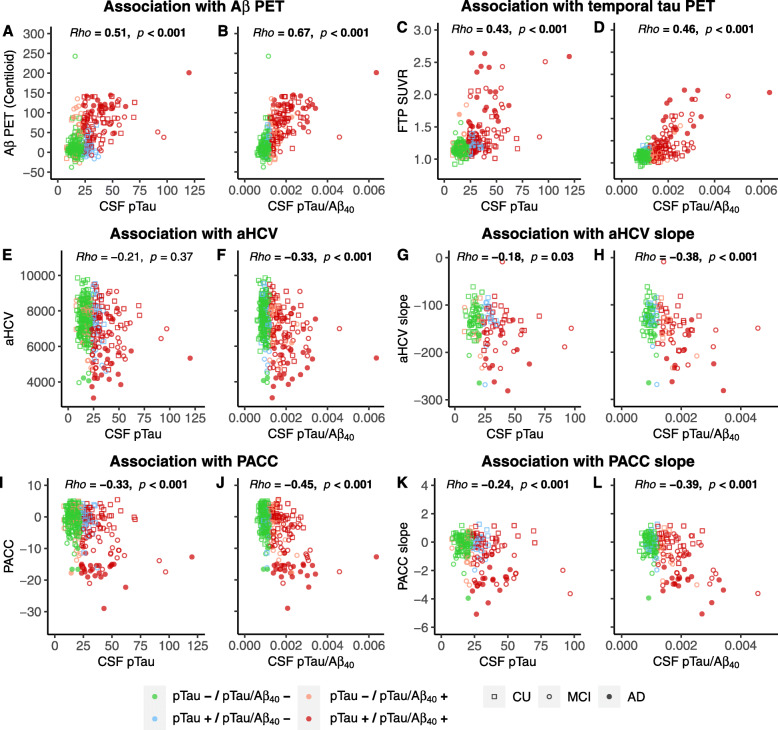
Associations between CSF pTau and pTau/Aβ_40_, Aβ PET, tau PET, neurodegeneration and cognition. Associations of baseline CSF pTau and pTau/Aβ_40_ with baseline Aβ PET (**a**, **b**), baseline Temporal-metaROI FTP SUVR (**c**, **d**), baseline (**e**, **f**), and slope (**g**, **h**) of adjusted hippocampal volume (aHCV) (mm^3^), baseline (**i**, **j**) and slope (**k**, **l**) of PACC cognitive score. Different colors reflect the concordance and discordance between CSF pTau and CSF pTau/Aβ_40_. For example, pTau−/pTau/Aβ_40_− indicates the individual was negative according to both CSF pTau and CSF pTau/Aβ_40_, while pTau+/pTau/Aβ_40_− indicates the individual was positive according to CSF pTau but negative according to CSF pTau/Aβ_40_

The slopes of FTP SUVR, aHCV, and PACC post baseline CSF collection were calculated using LME models over time from the first measurement point post baseline CSF collection (time = 0) to the last measurement point for each participant. The time variable is anchored to the baseline CSF measurement. In order to study whether elevated CSF pTau/Aβ_40_ is more related to the progression of AD than high CSF pTau, we also used GLM models to investigate the associations of CSF pTau and pTau/Aβ_40_ with Aβ PET, tau PET, aHCV, ΔaHCV, PACC, and ΔPACC, controlling for APOE-ε4 status, diagnosis, sex, age, and education. Since there was a time difference between baseline CSF collection point and the first measurements of FTP SUVR, aHCV, and PACC post baseline CSF collection, we included these time differences in the GLM models. Because we found use of the CSF pTau/Aβ_40_ ratio abolished the positive correlation between CSF pTau and Aβ_42_ among Aβ PET− range (see Fig. 1c, d in “Results”) and improved the associations with Aβ PET, tau PET, aHCV, ΔaHCV, PACC, and ΔPACC (see Fig. 2 in “Results”), we used this ratio in subsequent analyses.

We then explored the biological plausibility of the CSF pTau/Aβ_40_ by examining associations between CSF pTau/Aβ_40_ and FTP SUVRs in 35 Freesurfer-defined ROIs, controlling for Aβ PET (in Centiloids), APOE-ε4 status, diagnosis, sex, and age. Spearman’s rho was calculated between CSF pTau/Aβ_40_ and FTP SUVR. Subsequently, we examined the associations between Aβ PET, CSF pTau/Aβ_40_, CSF pTau, and tau PET (entorhinal or Temporal-metaROI) in Aβ− and Aβ+ participants, controlling for APOE-ε4 status, diagnosis, sex, and age.

In order to investigate the predictive effect of baseline Aβ PET, CSF pTau/Aβ_40_, and FTP on subsequent ΔFTP and ΔPACC, we used these variables at baseline to predict subsequent ΔFTP and ΔPACC in participants with longitudinal tau PET and PACC data respectively. In order to explore temporal relationships between Aβ and tau, we also examined the sequential associations between baseline Aβ PET, CSF pTau/Aβ_40_ ratio, FTP, and ΔFTP in Aβ+ participants using latent variable modeling (R; Lavaan package) [39].

For GLM models with non-Gaussian distribution outcomes (Aβ and tau PET), we used a “log” link function in the Gaussian family to study the associations between predictor and outcome. Spearman’s rank correlation coefficient (rho) was calculated between predictor and outcome. We selected *p* <  0.05 as the significance level. All statistical analyses were performed in the statistical program *R* (v3.6.2, The R Foundation for Statistical Computing).

## Results

### Demographics

Measurements were acquired between September 21, 2015 and April 9, 2020. Demographics can be found in Table 1. In total, 341 participants had contemporaneous CSF Aβ_40_, Aβ_42_ and pTau, Aβ PET, tau PET, structural MRI, and PACC cognitive score. At baseline, Aβ+ participants were significantly older and had greater CSF pTau, CSF pTau/Aβ_40_ and Temporal-metaROI FTP SUVR, lower aHCV, lower cognitive test scores, and a higher percentage of APOE-ε4 carriers than Aβ− participants. Longitudinally, 116, 139, and 202 participants had > 2 FTP PET scans (median follow-up 1.2 (range 0.7–3.3) years), structural MRI scans (median follow-up 1.4 (range 0.8–3.8) years), and PACC cognitive scores (median follow-up 1.2 (range 0.7–4.0) years) respectively.

**Table 1:** Characteristics of participants in this study

Aβ PET status	Aβ−	Aβ+	*p* value
**341 participants with CSF Aβ**_**40**_**, Aβ**_**42**_ **and pTau, Aβ PET, and tau PET**			
Sample size	195 (57%)	146 (43%)	
CU/MCI/AD	145/46/4	74/45/27	
Age (years)	70.4 (9.4)	74.7 (10.4)	**< 0.001**
Education (years)	18 (2)	16 (3)	0.07
Female (%)	115 (59%)	78 (53%)	0.44
APOE-ε4 (%)	37 (19%)	83 (57%)	**< 0.001**
Aβ PET (Centiloids)	4.9 (11.0)	71.2 (59.0)	**< 0.001**
CSF Aβ_42_	1421 (817)	653 (377)	**< 0.001**
CSF Aβ_40_	18,440 (7680)	17,770 (6150)	0.56
CSF pTau	17.8 (8.2)	27.2 (19.9)	**< 0.001**
CSF pTau/Aβ_40_	0.0010 (0.0002)	0.0016 (0.0009)	**< 0.001**
FTP SUVR (Temporal-metaROI)	1.16 (0.08)	1.28 (0.27)	**< 0.001**
aHCV (mm^3^)	7530 (1469)	6990 (1750)	**< 0.001**
PACC	0.25 (5.06)	−2.33 (11.64)	**< 0.001**
**116 participants with ≥ 2 tau PET scans**			
Sample size	41 (35%)	75 (65%)	
CU/MCI/AD	26/14/1	39/25/11	
FTP visits (median (IQR, range), no.)	2.0 (1.0, 2–4)	2.0 (1.0, 2–4)	
FTP follow-up (Median (IQR, range), years)	1.8 (1.1, 0.8–3.3)	1.2 (1.0, 0.7–3.1)	
**139 participants with ≥ 2 aHCV data**			
Sample size	64 (46%)	75 (54%)	
CU/MCI/AD	42/20/2	39/24/12	
MRI visits (median (IQR, range), no.)	2.0 (0, 2–4)	2.0 (0.5, 2–4)	
MRI follow-up (median (IQR, range), years)	2.0 (1.0, 0.9–3.8)	1.2 (0.9, 0.8–3.2)	
**202 participants with ≥ 2 PACC measurements**			
Sample size	99 (49%)	103 (51%)	
CU/MCI/AD	60/37/2	49/36/18	
PACC visits (median (IQR, range), no.)	2 (0, 2–4)	2 (1, 2–5)	
PACC follow-up (median (IQR, range), years)	2.0 (1.0, 0.9–3.0)	1.1 (1.0, 0.7–4.0)	

Abbreviations: *Aβ* amyloid-β, *AD* Alzheimer’s disease, *aHCV* adjusted hippocampal volume, *CU* cognitively unimpaired, *FTP* ^18^F-flortaucipir, *IQR* interquartile range, *MCI* mild cognitive impairment, *PACC* Preclinical Alzheimer Cognitive Composite, *pTau* phosphorylated tau, *SUVR* standardized uptake value ratio

### Use of CSF Aβ_40_ to adjust CSF pTau

CSF Aβ_40_ was not associated with Aβ PET or tau PET regardless of Aβ PET status (Fig. 1a, b). Before normalizing to CSF Aβ_40_, CSF pTau was positively (standardized *β* (*β*_std_) = 0.59[95% confidence interval (CI), 0.48, 0.71]) associated with CSF Aβ_42_ in Aβ PET− participants, whereas no association was found in Aβ+ participants (Fig. 1c). We also verified that there was a similar positive association between CSF pTau and CSF Aβ_42_ analyzed with mass spectrometry rather than the Roche Elecsys immunoassay in a partially overlapping (9.8%) sample of 384 Aβ− participants (Supplemental Fig. 9). After normalizing CSF pTau using CSF Aβ_40_, CSF pTau/Aβ_40_ was negatively (Fig. 1d) associated with CSF Aβ_42_ in both Aβ− (*β*_std_ = − 0.27 [95% CI, − 0.41, − 0.13]) and Aβ+ (*β*_std_ = − 0.32 [95% CI, − 0.48, − 0.15]) participants.

Notably, the association with Aβ PET increased from rho value 0.51 when using CSF pTau alone to 0.67 using the CSF pTau/Aβ_40_ (Fig. 2a, b). Likewise, the association with tau PET increased from rho value 0.43 when using CSF pTau alone to 0.46 using the CSF pTau/Aβ_40_ (Fig. 2c, d). We also compared CSF pTau and CSF pTau/Aβ_40_ in terms of their associations with other measures of neurodegeneration biomarkers and cognition in order to further investigate the validity of CSF pTau/Aβ_40_. CSF pTau/Aβ_40_ but not CSF pTau was negatively associated with baseline aHCV (Fig. 2e, f), and the association with aHCV slope increased from rho value − 0.18 when using CSF pTau alone to − 0.38 using the CSF pTau/Aβ_40_ (Fig. 2g, h). The association with baseline PACC and PACC slope increased from rho values − 0.33 and − 0.24 when using CSF pTau alone to − 0.45 and − 0.39 using the CSF pTau/Aβ_40_ respectively (Fig. 2i, l).

Based on these findings, CSF pTau/Aβ_40_ was used to represent tauopathy in CSF instead of CSF pTau for all subsequent analyses.

We also found that CSF pTau and CSF pTau/Aβ_40_ were both more strongly associated with Aβ PET than they were with tau PET (Fig. 2a–d).

### Regions with significant associations between CSF pTau/Aβ_40_ and tau PET

**Fig. 3:**
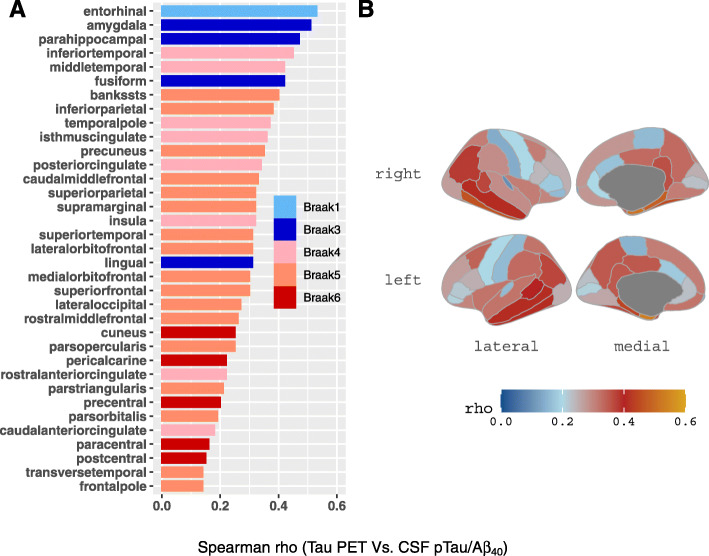
Regions with significant association between CSF pTau/Aβ_40_ and tau PET. **a** Correlation coefficients between CSF pTau/Aβ_40_ and tau PET SUVRs in Freesurfer-defined regions were illustrated in **a** bar graph and **b** brain map. Abbreviations: Aβ = amyloid-β; FTP = ^18^F-flortaucipir; pTau = phosphorylated tau; Spearman R = Spearman’s correlation coefficient; SUVR = standardized uptake value ratio

CSF pTau/Aβ_40_ was significantly associated with tau PET SUVRs in all the 35 ROIs, and the strongest association regions were within the Temporal-metaROI region (Fig. 3). We repeated these analyses in Aβ−, Aβ+, CU, and non-demented (CU and MCI) participants. The results were similar for Aβ+ participants (supplemental Fig. 10A), whereas no association was found for Aβ− participants. Similar features were observed for CU and non-demented (CU and MCI) participants (supplemental Fig. 10B-C). Because the strongest associations between CSF pTau/Aβ_40_ and tau PET were within the Temporal-metaROI (Fig. 3), which has been commonly used to detect tau deposition in brain [40–46], temporal tau PET (Temporal-metaROI FTP SUVR) was selected to represent tau deposition for further analyses unless otherwise noted.

### Cross-sectional associations between Aβ PET, CSF pTau/Aβ_40_, and tau PET

**Fig. 4:**
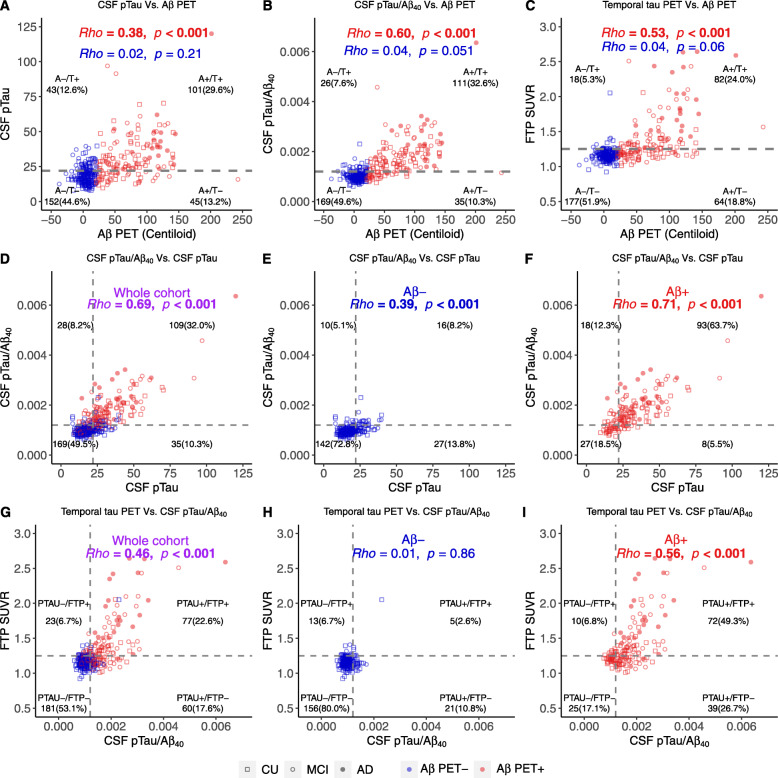
Cross-sectional associations between Aβ PET, CSF pTau/Aβ_40_, and tau PET. Associations between baseline Aβ PET and **a** CSF pTau, **b** CSF pTau/Aβ_40_, and **c** temporal tau PET. Associations between baseline CSF pTau and CSF pTau/Aβ_40_ in the whole cohort (**d**), Aβ− (**e**), and Aβ+ (**f**) participants. Associations between baseline CSF pTau/Aβ_40_ and Temporal-metaROI tau PET in the whole cohort (**g**), Aβ− (**h**), and Aβ+ (**i**) participants. The vertical and horizontal gray dashed lines reflect the abnormal thresholds of corresponding biomarkers in the *x*-axis and *y*-axis respectively. Abbreviations: Aβ = amyloid-β; A = Aβ PET; − = negative; + = positive; AD = Alzheimer’s disease; CU = cognitively unimpaired; FTP = ^18^F-flortaucipir; MCI = mild cognitive impairment; pTau = phosphorylated tau; PTAU = CSF pTau or CSF pTau/Aβ_40_ ratio; SUVR = standardized uptake value ratio; T = CSF pTau or CSF pTau/Aβ_40_ or tau PET

We found Aβ PET was significantly associated with CSF and PET tau measurements, which were driven by Aβ+ individuals. Baseline Aβ PET was positively associated with CSF pTau (Fig. 4a, *β*_std_ = 0.32 [95% CI, 0.15, 0.48]), CSF pTau/Aβ_40_ (Fig. 4b, *β*_std_ = 0.43 [95% CI, 0.28, 0.58]), and tau PET in Temporal-metaROI (Fig. 4c, *β*_std_ = 0.34 [95% CI, 0.21, 0.48]) and entorhinal (Supplemental Fig. 11A, *β*_std_ = 0.36 [95% CI, 0.23, 0.48]) in Aβ+ participants. Notably, the association with Aβ PET increased from rho value 0.38 when using CSF pTau alone to 0.60 using the CSF pTau/Aβ_40_ (Fig. 4a)_._ In Aβ− participants, Aβ PET was weakly but significantly associated with tau PET in entorhinal (Supplemental Fig. 11A, *β*_std_ = 0.17 [95% CI, 0.02, 0.33]).

In order to investigate the prevalence of abnormal CSF pTau, CSF pTau/Aβ_40_, and tau PET (entorhinal or Temporal-metaROI), Aβ− and Aβ+ participants were classified as tau normal (T−)/abnormal (T+) using CSF pTau or CSF pTau/Aβ_40_ or tau PET thresholds, dividing the whole cohort into A−/T−, A−/T+, A+/T−, and A+/T+ groups. Few Aβ− participants had abnormal CSF pTau/Aβ_40_ (7.6%) and temporal tau PET (5.3%), whereas Aβ+ participants showed a 3.0–4.5 times higher percentage of abnormal CSF pTau/Aβ_40_ (32.6%) and temporal tau PET (24.0%) than Aβ− participants (Fig. 4b, c). Among Aβ− participants, abnormal CSF pTau had 1.66 times (12.6% vs. 7.6%) higher prevalence than abnormal CSF pTau/Aβ_40_ (Fig. 4a, b). The results were similar for entorhinal tau PET (Supplemental Fig. 11A).

In order to determine the concordance between CSF pTau and CSF pTau/Aβ_40_, and between CSF and PET measures of tau, participants were classified as normal (−)/abnormal (+) on CSF pTau or CSF pTau/Aβ_40_ (PTAU+/−) and entorhinal or Temporal-metaROI FTP SUVR (FTP+/−). Abnormal CSF pTau only had higher prevalence (Fig. 4e, 13.8% vs. 5.1%, odds ratio = 2.7[95%CI, 1.3–6.3], *p* = 0.008) than abnormal CSF pTau/Aβ_40_ only in Aβ− participants, whereas abnormal CSF pTau/Aβ_40_ only had marginally higher prevalence (Fig. 4f, 12.3% vs. 5.5%, odds ratio = 2.3[95%CI, 0.9–6.0], *p* = 0.08) than abnormal CSF pTau only in Aβ+ participants. CSF pTau/Aβ_40_ (Fig. 4g, *β*_std_ = 0.59 [95% CI, 0.51, 0.68]) were positively associated with temporal tau PET across all participants. Aβ+ participants were responsible for this relationship because no association was found in Aβ− participants (Fig. 4h, i). We found that in Aβ− participants, the proportion of participants with abnormal CSF pTau/Aβ_40_ only was comparable to those with an abnormal temporal tau PET only (10.8% vs. 6.7%) (Fig. 4h). In contrast, in Aβ+ participants, those with abnormal CSF pTau/Aβ_40_ only were fourfold more prevalent than the abnormal temporal tau PET only (Fig. 4i, 26.7% vs. 6.8%, odds ratio = 3.9[95%CI, 1.9–8.8], *p* <  0.001). The results were similar for entorhinal tau PET (Supplemental Fig. 11B-D).

The conservative cutoffs of CSF pTau, CSF pTau/Aβ_40_, entorhinal tau PET, and temporal tau PET were higher and defined fewer “T+” individuals, while the results of concordance of different biomarkers were substantially the same as the initial cutoffs (Supplemental Figs. 12–13).

### Associations between Aβ PET, CSF pTau/Aβ_40_, tau PET and longitudinal tau PET change

**Fig. 5:**
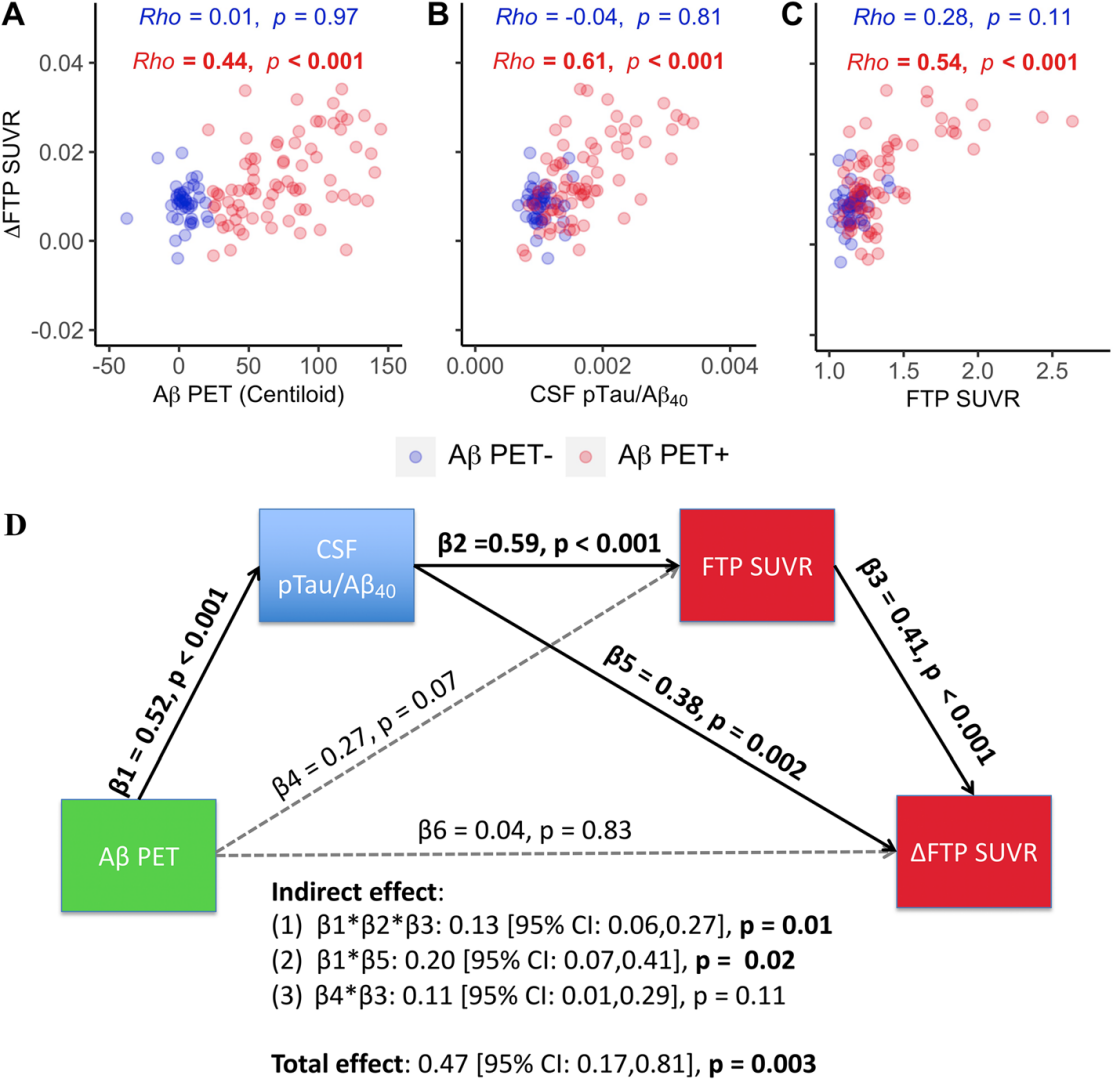
Associations between baseline Aβ PET, CSF pTau/Aβ_40_, tau PET and longitudinal tau PET change. Associations between annual temporal tau PET change (ΔFTP SUVR in Temporal-metaROI) and **a** baseline Aβ PET, **b** CSF pTau/Aβ_40_, and **c** Temporal-metaROI tau PET (FTP SUVR in Temporal-metaROI). **d** All the possible pathways between Aβ PET, CSF pTau/Aβ_40_, FTP, and ΔFTP were calculated in a serial mediation model in Aβ+ participants. Aβ PET, CSF pTau/Aβ_40_, FTP, and ΔFTP were converted to standard *z* scores. Total, direct, and indirect associations were calculated via a 5000-iteration bootstrapping procedure. Abbreviations: Aβ = amyloid-β; FTP = ^18^F-flortaucipir; pTau = phosphorylated tau; SUVR = standardized uptake value ratio

Baseline Aβ PET (Fig. 5a, *β*_std_ = 0.42 [95% CI, 0.22, 0.63]), CSF pTau/Aβ_40_ (Fig. 5b, *β*_std_ = 0.61 [95% CI, 0.43, 0.79]), and Temporal-metaROI tau PET (Fig. 5c, *β*_std_ = 0.63 [95% CI, 0.45, 0.81]) were all associated with subsequent tau PET increase (ΔFTP) in Aβ+ participants (Fig. 5a–c). In contrast, no predictive effect was found in Aβ− participants.

The latent variable model demonstrated that the direct association between Aβ and ΔFTP increase in Aβ+ participants was not significant after including the CSF pTau/Aβ_40_ and FTP (Fig. 5d), reducing the *β* value from 0.47 to 0.04 (91% change). CSF pTau/Aβ_40_-involved pathways (pathway1: from Aβ PET to CSF pTau/Aβ_40_ to ΔFTP; pathway2: from Aβ PET to CSF pTau/Aβ_40_ to FTP to ΔFTP) explained 70% of the association (total effect) between Aβ PET and ΔFTP increase in Aβ+ participants.

### Prediction of longitudinal cognitive decline

**Fig. 6:**
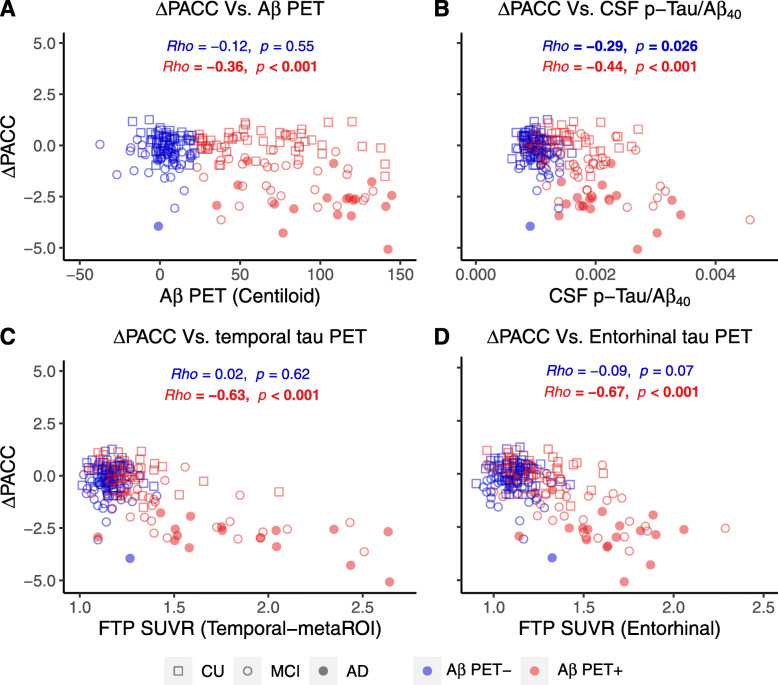
Associations between longitudinal cognitive decline and baseline Aβ PET, CSF pTau/Aβ_40_, and tau PET. Associations between annual PACC change (ΔPACC) and baseline **a** Aβ PET (Centiloid), **b** CSF pTau/Aβ_40_ ratio, **c** temporal tau PET (Temporal-metaROI FTP SUVR), and **d** entorhinal tau PET (entorhinal FTP SUVR). Abbreviations: Aβ = amyloid-β; AD = Alzheimer’s disease; CU = cognitively unimpaired; FTP = ^18^F-flortaucipir; MCI = mild cognitive impairment; PACC = Preclinical Alzheimer Cognitive Composite; pTau = phosphorylated tau; SUVR = standardized uptake value ratio

Baseline Aβ PET (Fig. 6a, *β*_std_ = − 0.41 [95% CI, − 0.59, − 0.23]), CSF pTau/Aβ_40_ (Fig. 6b, *β*_std_ = − 0.53 [95% CI, − 0.69, − 0.36]), and Temporal-metaROI tau PET (Fig. 6c, *β*_std_ = − 0.73 [95% CI, − 0.86, − 0.60]) were all associated with subsequent cognitive decline in Aβ+ participants (Fig. 6), whereas only tau PET (*β*_std_ = − 0.68[95% CI, − 0.87, − 0.48], *p* <  0.001) remained predictive when all variables were added into one multivariate model. The results were similar for entorhinal tau PET. In contrast, only CSF pTau/Aβ_40_ (*β*_std_ = − 0.22[95% CI, − 0.42, − 0.03], *p* = 0.03) was associated with subsequent cognitive decline in Aβ− participants.

## Discussion

This study had several primary findings: (1) use of a CSF pTau/Aβ_40_ ratio reduced noise in pTau likely introduced by individual variability in CSF production rates, and increased associations with Aβ PET, tau PET, hippocampal volume, and cognition compared with CSF pTau alone. (2) Tau PET associations with CSF pTau/Aβ_40_ were highest in medial and lateral temporal regions. (3) Associations between Aβ PET, CSF pTau/Aβ_40,_ and tau PET (cross-sectionally and longitudinally) were substantially driven by Aβ PET-positive individuals. (4) Among these Aβ+ individuals, most participants (66%) were concordant on CSF pTau/Aβ_40_ and Temporal-metaROI tau PET, but among discordant individuals, those with abnormal CSF pTau/Aβ_40_ and normal tau PET were 4 times more prevalent (26.7%) than those with abnormal tau PET and normal CSF pTau/Aβ_40_ (6.8%). (5) Among these Aβ+ individuals, baseline Aβ PET, CSF pTau/Aβ_40_, and tau PET were all associated with subsequent tau PET increase, while CSF pTau/Aβ_40_ significantly mediates the association between Aβ PET and tau PET (cross-sectionally and longitudinally). (6) Only tau PET was predictive of longitudinal cognitive decline when baseline Aβ PET, CSF pTau/Aβ40, and tau PET were put in one multivariate model.

Our motivation to adjust CSF pTau measurements was based on our observation that Aβ PET-negative individuals had abnormal (“positive”) CSF pTau that correlated positively with high (“normal”) CSF Aβ_42_ (Fig. 1c), suggesting that these elevated measurements reflect high CSF total production rate but not abnormal tau. Similar patterns of elevated pTau and CSF Aβ_42_ in the negative range that are presumably artifactual have been observed in other recent studies from ADNI, BIOFINDER, and Washington University [7, 16], and with CSF data analyzed with mass spectrometry (Supplementary Fig. 9) and immunoassays. CSF pTau/Aβ_40_ appears to be a compelling strategy for improving sensitivity to CSF tau pathology, since this approach reversed the biologically implausible association between CSF pTau and Aβ_42_ and improved associations with downstream markers of AD progression compared with CSF pTau alone. Because CSF Aβ_40_ was not associated with PET measures of either Aβ or tau (Fig. 1a, b) and is not elevated in AD [21–29], its use as a normalization variable is unlikely to bias estimates of CSF pTau. This strategy is in line with recent work supporting use of CSF Aβ_42_/Aβ_40_ instead of CSF Aβ_42_ alone [6, 7, 17–19], and use of CSF pTau/tTau instead of CSF pTau [47]. However, our results did not exclude other possibilities for the enhanced associations between CSF pTau/Aβ_40_ and downstream markers of AD progression. For example, a few studies [48–51] have reported that CSF Aβ_40_ may decrease in cognitively impaired individuals, which may thereby increase the CSF pTau/Aβ_40_ ratios of cognitively impaired individuals. In addition, one animal study [52] observed that CSF Aβ_40_ may increase in the earliest phase of Aβ accumulation in mouse models, which may delay the increase of CSF pTau/Aβ_40_ in the preclinical stage of AD. We found only trend-level decreases in CSF Aβ_40_ in Aβ− unimpaired and Aβ+ impaired groups relative to Aβ+ unimpaired individuals (data not shown), but it is possible that early and late changes in CSF Aβ_40_ may contribute to the tau-related effects we observed.

Associations between CSF pTau/Aβ_40_ and tau PET were stronger in ROIs in the temporal lobe than other areas such as frontal and occipital lobes that accumulate tau in later stages of disease [53, 54], consistent with our observation and recent studies [14, 15, 55] that CSF tauopathy is an early marker of tau pathology. The strongest associations were within the medial and lateral temporal regions that overlapped with a tau composite region (Temporal-metaROI) reported previously as well as a “Braak III/IV” like ROI [40, 41, 45, 56]. Notably, the relationship between CSF pTau/Aβ_40_ and tau PET was primarily driven by Aβ PET positivity and less influenced by clinical diagnosis (Supplementary Fig. 10), which could also reflect a greater range of tau pathology in Aβ+ individuals and a stronger relationship between Aβ and tau than between tau and clinical symptoms [57, 58]. Consistent with the present study, Chhatwal et al. [10] reported a significant association between CSF pTau and tau PET in limbic regions of the temporal lobe in CU elderly adults. However, two studies [9, 12] did not find significant association between CSF pTau and tau PET in CU individuals, perhaps due to methodological factors such as sample size and the use of CSF pTau alone rather than the CSF pTau/Aβ_40_ ratio.

Elevated Aβ PET was weakly associated with greater tau (CSF pTau/Aβ_40_ or tau PET) in the Aβ− individuals, which was in line with previous reports [59–62]. However, also consistent with previous studies [42, 63, 64], we found that tau (CSF pTau/Aβ_40_ or tau PET) was rarely (5.3–7.9%) abnormal in the Aβ− range (Fig. 4). Furthermore, baseline Aβ PET, CSF pTau/Aβ_40_, and tau PET were predictive of subsequent tau PET increase in the Aβ+ group only, which is in agreement with recent tau PET studies [40, 65]. Together, these findings suggest that tau is rarely increasing or abnormal when Aβ is absent.

In line with our findings, one recent study [15] also reported that CSF pTau mediated the association between Aβ PET and tau PET, and higher CSF pTau was associated with faster tau PET increase rates in cognitively impaired individuals. Unlike this study, we found baseline tau PET was also related to the tau PET rate. The discrepancy may be explained by the larger sample size and the use of white matter reference for longitudinal tau PET in the present study. In the mediation analyses, two significant CSF pTau/Aβ_40_-linked pathways were identified, which explained 70% of the association between Aβ PET and longitudinal brain tau accumulation among Aβ+ individuals.

Finally, consistent with three recent reports [14, 15, 66], we found that tau PET was more predictive of subsequent cognitive decline than CSF tau among Aβ+ individuals, suggesting brain tau may reflect a later tau stage closer to cognitive decline than CSF tau on the Alzheimer’s continuum. Interestingly, previous comparisons of CSF and PET measurements of Aβ were analogous in showing that cognitive decline is more related to Aβ PET than CSF Aβ [1, 3, 67, 68]. We also noticed that higher CSF pTau/Aβ_40_ was significantly related to faster longitudinal cognitive decline in amyloid-negative individuals. No previous studies reported the association between CSF pTau and cognitive decline in amyloid-negative individuals, which should be cautious to interpret this result and may need to be validated in other samples.

This study has several limitations. The CSF pTau/Aβ_40_ threshold was derived from the existing sample of ADNI participants and only pTau_181_ was available in the ADNI sample at this time, so it would be helpful to validate the findings in other samples and with other phosphorylation sites (i.e., pTau_217_ [47, 69]) and tau PET ligands. Furthermore, only 9% (31/341) of the participants in this study were AD patients and the longitudinal observation was of relatively short duration, so it would be helpful to confirm those findings using additional participants and extended longitudinal data. Finally, one possible explanation for the differences we observed between tau PET and CSF pTau measurements is that CSF pTau may reflect Aβ in addition to tau pathology. Our observation that both CSF pTau and CSF pTau/Aβ_40_ had stronger associations with Aβ PET than they did with tau PET (Fig. 2a–d) is consistent with this possibility, but further pathology studies are needed to verify this interpretation.

## Conclusions

In summary, we found that the use of a CSF pTau/Aβ_40_ ratio improves the sensitivity to detect CSF tau by adjusting for individual differences in CSF production. Furthermore, although PET and CSF measures of tau are broadly concordant in the majority (76%) of individuals when measured dichotomously, our findings support recent work [14] indicating that CSF and PET measures of tau may not be interchangeable in the A/T/N research framework [70]. Among amyloid-positive individuals, higher tauopathy measured with CSF and PET is related to faster tau accumulation, while tau PET was more predictive of subsequent cognitive decline than CSF tau. Taken together, these findings suggest that the interchangeability of PET and CSF measures of tau likely depends on the goals of the study, the phase of AD being studied, and the clinical characteristics of the population.

## Supplementary information

**Additional file 1:** **Figure S1.** The ROC analysis using the Youden index classifying 280 Aβ- ADNI cognitively unimpaired (CU) participants and 183 Aβ + ADNI MCI and AD patients as the endpoint to define the cutoff ≥1.25 for Temporal-metaROI FTP SUVR. AUC: 0.876 (95%CI, 0.84, 0.912). Among these 463 ADNI participants, 217 (47%) participants were included in the analyses of the manuscript. **Figure S2.** Histograms of Temporal-metaROI FTP SUVRs of (A) all 775 ADNI participants, (B) 280 Aβ- ADNI CU participants and (C) 183 Aβ + ADNI MCI and AD patients with tau PET scan. Red dotted line is the cutoff of Temporal-metaROI FTP SUVR 1.25. **Figure S3.** The ROC analysis using the Youden index classifying 280 Aβ- ADNI CU participants and 183 Aβ + ADNI MCI and AD patients as the endpoint to define the cutoff ≥1.21 for entorhinal FTP SUVR. AUC: 0.891 (95%CI, 0.856, 0.926). **Figure S4.** Histograms of entorhinal FTP SUVRs of (A) all 775 ADNI participants, (B) 280 Aβ- ADNI CU participants and (C) 183 Aβ + ADNI MCI and AD patients with tau PET scan. Red dotted line is the cutoff of entorhinal FTP SUVR 1.21. **Figure S5.** The ROC analysis using the Youden index classifying 320 Aβ- ADNI CU participants and 429 Aβ + ADNI MCI and AD patients as the endpoint to define the cutoff ≥22 for CSF p-Tau. AUC: 0.865 (95%CI, 0.84, 0.89). Among these 749 ADNI participants, 212 (28%) participants were included in the analyses of the manuscript. **Figure S6.** Histograms of CSF p-Tau of (A) all 1534 ADNI participants, (B) 320 Aβ- ADNI CU participants and (C) 429 Aβ + ADNI MCI and AD patients with CSF p-Tau measurement. Red dotted line is the cutoff of CSF p-Tau 22. **Figure S7.** The ROC analysis using the Youden index classifying 169 Aβ- ADNI CU participants and 160 Aβ + ADNI MCI and AD patients as the endpoint to define the cutoff ≥0.0012 for CSF p-Tau/Aβ_40_ ratio. AUC: 0.976 (95%CI, 0.96, 0.99). Among these 329 ADNI participants, 201 (61%) participants were included in the analyses of the manuscript. **Figure S8.** Histograms of CSF p-Tau/Aβ_40_ for (A) all 447 ADNI participants, (B) 169 Aβ- ADNI CU participants and (C) 160 Aβ + ADNI MCI and AD patients with CSF p-Tau/Aβ_40_. Red dotted line is the 0.0012 cutoff for the CSF p-Tau/Aβ_40_ ratio. **Figure S9.** Cross-sectional associations between CSF MASS Aβ_42_ and CSF p-Tau. The vertical gray dashed line reflects the abnormal threshold of CSF p-Tau. Abbreviations: p-Tau = phosphorylated tau; Aβ = amyloid-β; CU = cognitively unimpaired; MCI = mild cognitive impairment; AD = Alzheimer’s disease. **Figure S10.** Regions with significant association between CSF P-tau and FTP tau in (A) Aβ+, (B) CU and (C) non-demented participants. Abbreviations: Spearman rho = Spearman’s correlation coefficient; p-Tau = phosphorylated tau; Aβ = amyloid-β; FTP = ^18^F-flortaucipir; SUVR = standardized uptake value ratio; CU = cognitively unimpaired; MCI = mild cognitive impairment; AD = Alzheimer’s disease. **Figure S11.** Cross-sectional associations between Aβ PET, CSF p-Tau/Aβ_40_ and entorhinal tau PET. (A). Associations between baseline entorhinal tau PET and Aβ PET. Associations between baseline CSF p-Tau/Aβ_40_ and entorhinal tau PET in the whole cohort (B), Aβ- (C) and Aβ + (D) participants. The vertical and horizontal gray dashed lines reflect the abnormal thresholds of corresponding biomarkers in x-axis and y-axis respectively. Abbreviations: Aβ = amyloid-β; A = Aβ PET; − = negative; + = positive; AD = Alzheimer’s disease; CU = cognitively unimpaired; FTP = ^18^F-flortaucipir; MCI = mild cognitive impairment. **Figure S12.** Cross-sectional associations between Aβ PET, CSF pTau/Aβ_40_ and tau PET using alternative cutoffs. Associations between baseline Aβ PET and (A) CSF pTau, (B) CSF pTau/Aβ_40_ and (C) temporal tau PET. Associations between baseline CSF pTau and CSF pTau/Aβ_40_ in the whole cohort (D), Aβ- (E) and Aβ + (F) participants. Associations between baseline CSF pTau/Aβ_40_ and Temporal-metaROI tau PET in the whole cohort (G), Aβ- (H) and Aβ + (I) participants. The vertical and horizontal gray dashed lines reflect the abnormal thresholds of corresponding biomarkers in x-axis and y-axis respectively. Abbreviations: Aβ = amyloid-β; A = Aβ PET; − = negative; + = positive; AD = Alzheimer’s disease; CU = cognitively unimpaired; FTP = ^18^F-flortaucipir; MCI = mild cognitive impairment; pTau = phosphorylated tau; PTAU = CSF pTau or CSF pTau/Aβ_40_ ratio; SUVR = standardized uptake value ratio; T = CSF pTau or CSF pTau/Aβ_40_ or tau PET. **Figure S13.** Cross-sectional associations between Aβ PET, CSF p-Tau/Aβ_40_ and entorhinal tau PET using alternative cutoffs. (A). Associations between baseline entorhinal tau PET and Aβ PET. Associations between baseline CSF p-Tau/Aβ_40_ and entorhinal tau PET in the whole cohort (B), Aβ- (C) and Aβ + (D) participants. The vertical and horizontal gray dashed lines reflect the abnormal thresholds of corresponding biomarkers in x-axis and y-axis respectively. Abbreviations: Aβ = amyloid-β; A = Aβ PET; − = negative; + = positive; AD = Alzheimer’s disease; CU = cognitively unimpaired; FTP = ^18^F-flortaucipir; MCI = mild cognitive impairment.

## Data Availability

The dataset supporting the conclusions of this article is available in the ADNI repository (ida.loni.usc.edu). Derived data is available from the corresponding author on request by any qualified investigator subject to a data use agreement.

## Abbreviations

Aβ: Amyloid-β
ADNI: Alzheimer’s Disease Neuroimaging Initiative
*β*_std_: Standardized β coefficient
CSF pTau: CSF phosphorylated tau
CU: Cognitively unimpaired
CI: Confidence interval
FTP: ^18^F-flortaucipir
FBP: ^18^F-florbetapir
FBB: ^18^F-florbetaben
GLM: Generalized linear model
MCI: Mild cognitive impairment
PACC: Preclinical Alzheimer Cognitive Composite
ROI: Regions of interest
SNAP: Suspected non-Alzheimer’s pathology
SUVR: Standardized uptake value ratio
