# Supplementary material for: Normalization of CSF pTau measurement by Aβ40 improves its performance as a biomarker of Alzheimer’s disease
Source: Alzheimers Res Ther. 2020 Aug 15;12:97. doi: 10.1186/s13195-020-00665-8 (PMC7429887; doi:10.1186/s13195-020-00665-8)
Supplement: Supplementary file 1 — Additional file 1: Figure S1. The ROC analysis using the Youden index classifying 280 Aβ- ADNI cognitively unimpaired (CU) participants and 183 Aβ + ADNI MCI and AD patients as the endpoint to define the cutoff ≥1.25 for Temporal-metaROI FTP SUVR. AUC: 0.876 (95%CI, 0.84, 0.912). Among these 463 ADNI participants, 217 (47%) participants were included in the analyses of the manuscript. Figure S2. Histograms of Temporal-metaROI FTP SUVRs of (A) all 775 ADNI participants, (B) 280 Aβ- ADNI CU participants and (C) 183 Aβ + ADNI MCI and AD patients with tau PET scan. Red dotted line is the cutoff of Temporal-metaROI FTP SUVR 1.25. Figure S3. The ROC analysis using the Youden index classifying 280 Aβ- ADNI CU participants and 183 Aβ + ADNI MCI and AD patients as the endpoint to define the cutoff ≥1.21 for entorhinal FTP SUVR. AUC: 0.891 (95%CI, 0.856, 0.926). Figure S4. Histograms of entorhinal FTP SUVRs of (A) all 775 ADNI participants, (B) 280 Aβ- ADNI CU participants and (C) 183 Aβ + ADNI MCI and AD patients with tau PET scan. Red dotted line is the cutoff of entorhinal FTP SUVR 1.21. Figure S5. The ROC analysis using the Youden index classifying 320 Aβ- ADNI CU participants and 429 Aβ + ADNI MCI and AD patients as the endpoint to define the cutoff ≥22 for CSF p-Tau. AUC: 0.865 (95%CI, 0.84, 0.89). Among these 749 ADNI participants, 212 (28%) participants were included in the analyses of the manuscript. Figure S6. Histograms of CSF p-Tau of (A) all 1534 ADNI participants, (B) 320 Aβ- ADNI CU participants and (C) 429 Aβ + ADNI MCI and AD patients with CSF p-Tau measurement. Red dotted line is the cutoff of CSF p-Tau 22. Figure S7. The ROC analysis using the Youden index classifying 169 Aβ- ADNI CU participants and 160 Aβ + ADNI MCI and AD patients as the endpoint to define the cutoff ≥0.0012 for CSF p-Tau/Aβ40 ratio. AUC: 0.976 (95%CI, 0.96, 0.99). Among these 329 ADNI participants, 201 (61%) participants were included in the analyses of the manuscript. Figure S8. Histog [file 13195_2020_665_MOESM1_ESM.docx]

**Supplemental Material**

**Cutoff of tau PET**

***Supplemental fig.1.*** *The ROC analysis using the Youden index classifying 280 Aβ- ADNI cognitively unimpaired (CU) participants and 183 Aβ+ ADNI MCI and AD patients as the endpoint to define the cutoff ≥1.25 for Temporal-metaROI FTP SUVR. AUC: 0.876 (95%CI, 0.84, 0.912). Among these 463 ADNI participants, 217 (47%) participants were included in the analyses of the manuscript.*

***Supplemental fig.2.*** *Histograms of Temporal-metaROI FTP SUVRs of (A) all 775 ADNI participants, (B) 280 Aβ- ADNI CU participants and (C) 183 Aβ+ ADNI MCI and AD patients with tau PET scan. Red dotted line is the cutoff of Temporal-metaROI FTP SUVR 1.25.*

***Supplemental fig.3.*** *The ROC analysis using the Youden index classifying 280 Aβ- ADNI CU participants and 183 Aβ+ ADNI MCI and AD patients as the endpoint to define the cutoff ≥1.21 for entorhinal FTP SUVR. AUC: 0.891 (95%CI, 0.856, 0.926).*

***Supplemental fig.4.*** *Histograms of entorhinal FTP SUVRs of (A) all 775 ADNI participants, (B) 280 Aβ- ADNI CU participants and (C) 183 Aβ+ ADNI MCI and AD patients with tau PET scan. Red dotted line is the cutoff of entorhinal FTP SUVR 1.21.*

**Cutoff of CSF p-Tau**

***Supplemental fig.5.*** *The ROC analysis using the Youden index classifying 320 Aβ- ADNI CU participants and 429 Aβ+ ADNI MCI and AD patients as the endpoint to define the cutoff ≥22 for CSF p-Tau. AUC: 0.865 (95%CI, 0.84, 0.89). Among these 749 ADNI participants, 212 (28%) participants were included in the analyses of the manuscript.*

***Supplemental fig.6.*** *Histograms of CSF p-Tau of (A) all 1534 ADNI participants, (B) 320 Aβ- ADNI CU participants and (C) 429 Aβ+ ADNI MCI and AD patients with CSF p-Tau measurement. Red dotted line is the cutoff of CSF p-Tau 22.*

**Cutoff of CSF p-Tau/Aβ_40_ ratio**

***Supplemental fig.7.*** *The ROC analysis using the Youden index classifying 169 Aβ- ADNI CU participants and 160 Aβ+ ADNI MCI and AD patients as the endpoint to define the cutoff ≥0.0012 for CSF p-Tau/Aβ_40_ ratio. AUC: 0.976 (95%CI, 0.96, 0.99). Among these 329 ADNI participants, 201 (61%) participants were included in the analyses of the manuscript.*

***Supplemental fig.8.*** *Histograms of CSF p-Tau/Aβ_40_ for (A) all 447 ADNI participants, (B) 169 Aβ- ADNI CU participants and (C) 160 Aβ+ ADNI MCI and AD patients with CSF p-Tau/Aβ_40_. Red dotted line is the 0.0012 cutoff for the CSF p-Tau/Aβ_40_ ratio.*

**Association between CSF p-Tau and CSF Aβ_42_ analyzed with mass spectrometry**

***Supplemental fig.9.*** ***Cross-sectional associations between CSF MASS Aβ_42_ and CSF p-Tau*.** *The vertical gray dashed line reflects the abnormal threshold of CSF p-Tau. Abbreviations: p-Tau = phosphorylated tau;* *Aβ=amyloid-β; CU = cognitively unimpaired; MCI = mild cognitive impairment; AD = Alzheimer’s disease.*

We also investigated the association between CSF p-Tau and CSF Aβ42 analyzed with mass spectrometry rather than the Roche Elecsys immunoassay in 839 ADNI participants (Aβ-: n=384, Aβ+: n=455) who had concurrent CSF measurements with both Roche Elecsys immunoassay and 2D-UPLC-tandem mass spectrometry, and amyloid PET within 1 year. The CSF mass Aβ_42_ was calculated by the ADNI Biomarker core laboratory via 2D-UPLC-tandem mass spectrometry as described in a previous report[1]. We found similar positive association between CSF p-Tau and CSF mass Aβ_42_ in Aβ PET- individuals.

**Regions with significant associations between CSF p-Tau/Aβ_40_ ratio and tau PET in different Aβ status and diagnostic groups**

***Supplemental fig. 10.*** *Regions with significant association between CSF P-tau and FTP tau in (A) Aβ+, (B) CU and (C) non-demented participants. Abbreviations: Spearman rho = Spearman’s correlation coefficient; p-Tau = phosphorylated tau;* *Aβ=amyloid-β; FTP = ^18^F-flortaucipir; SUVR = standardized uptake value ratio; CU = cognitively unimpaired; MCI = mild cognitive impairment; AD = Alzheimer’s disease.*

**Cross-sectional associations between Aβ PET, CSF p-Tau/Aβ_40_ and entorhinal tau PET**

**

***Supplemental fig.11.*** ***Cross-sectional associations between Aβ PET, CSF p-Tau/Aβ_40_ and entorhinal tau PET****. (A). Associations between baseline entorhinal tau PET and Aβ PET. Associations between baseline CSF p-Tau/Aβ_40_* *and entorhinal tau PET in the whole cohort (B), Aβ- (C) and Aβ+(D) participants. The vertical and horizontal gray dashed lines reflect the abnormal thresholds of corresponding biomarkers in x-axis and y-axis respectively. Abbreviations: Aβ=amyloid-β; A = Aβ PET; - = negative; + = positive; AD = Alzheimer’s disease; CU = cognitively unimpaired; FTP = ^18^F-flortaucipir; MCI = mild cognitive impairment.*

**Concordance and discordance between CSF p-Tau, CSF p-Tau/Aβ_40_ and tau PET using alternative cutoffs**

For the alternative cutoffs of CSF p-Tau, CSF p-Tau/Aβ_40_ and, entorhinal and Temporal-metaROI FTP SUVRs as the mean + 2 SD of Aβ PET- CU ADNI individuals, the results were substantially the same.

***Supplemental fig.12.*** ***Cross-sectional associations between Aβ PET, CSF pTau/Aβ_40_ and tau PET using alternative cutoffs****. Associations between baseline Aβ PET and (A) CSF pTau, (B) CSF pTau/Aβ_40_ and (C) temporal tau PET. Associations between baseline CSF pTau* *and CSF pTau/Aβ_40_ in the whole cohort (D), Aβ- (E) and Aβ+(F) participants. Associations between baseline CSF pTau/Aβ_40_* *and Temporal-metaROI tau PET in the whole cohort (G), Aβ- (H) and Aβ+(I) participants. The vertical and horizontal gray dashed lines reflect the abnormal thresholds of corresponding biomarkers in x-axis and y-axis respectively. Abbreviations: Aβ=amyloid-β; A = Aβ PET; - = negative; + = positive; AD = Alzheimer’s disease; CU = cognitively unimpaired; FTP = ^18^F-flortaucipir; MCI = mild cognitive impairment; pTau = phosphorylated tau; PTAU = CSF pTau or CSF pTau/Aβ_40_ ratio; SUVR = standardized uptake value ratio; T = CSF pTau or CSF pTau/Aβ_40_ or tau PET.*

***Supplemental fig.13.*** ***Cross-sectional associations between Aβ PET, CSF p-Tau/Aβ_40_ and entorhinal tau PET using alternative cutoffs****. (A). Associations between baseline entorhinal tau PET and Aβ PET. Associations between baseline CSF p-Tau/Aβ_40_* *and entorhinal tau PET in the whole cohort (B), Aβ- (C) and Aβ+(D) participants. The vertical and horizontal gray dashed lines reflect the abnormal thresholds of corresponding biomarkers in x-axis and y-axis respectively. Abbreviations: Aβ=amyloid-β; A = Aβ PET; - = negative; + = positive; AD = Alzheimer’s disease; CU = cognitively unimpaired; FTP = ^18^F-flortaucipir; MCI = mild cognitive impairment.*

**Reference**

1. Korecka M, Waligorska T, Figurski M, Toledo JB, Arnold SE, Grossman M, et al. Qualification of a Surrogate Matrix-Based Absolute Quantification Method for Amyloid-β42 in Human Cerebrospinal Fluid Using 2D UPLC-Tandem Mass Spectrometry. J Alzheimer’s Dis. 2014;41:441–451.
